# Multispecies and Clonal Dissemination of OXA-48 Carbapenemase in *Enterobacteriaceae* From Companion Animals in Germany, 2009—2016

**DOI:** 10.3389/fmicb.2018.01265

**Published:** 2018-06-14

**Authors:** Sandra Pulss, Inka Stolle, Ivonne Stamm, Ursula Leidner, Carsten Heydel, Torsten Semmler, Ellen Prenger-Berninghoff, Christa Ewers

**Affiliations:** ^1^Institute of Hygiene and Infectious Diseases of Animals, Justus Liebig University Giessen, Giessen, Germany; ^2^Institute of Microbiology, University of Veterinary Medicine Hannover, Foundation, Hanover, Germany; ^3^Vet Med Labor GmbH, Division of IDEXX Laboratories, Ludwigsburg, Germany; ^4^Microbial Genomics (NG1), Robert Koch Institute, Berlin, Germany

**Keywords:** *Escherichia coli*, *Klebsiella pneumoniae*, *Enterobacter cloacae*, companion animals, carbapenemase, nosocomial

## Abstract

The increasing spread of carbapenemase-producing *Enterobacteriaceae* (CPE) poses a serious threat to public health. Recent studies suggested animals as a putative source of such bacteria. We investigated 19,025 *Escherichia coli*, 1607 *Klebsiella* spp. and 570 *Enterobacter* spp. isolated from livestock, companion animal, horse, and pet samples between 2009 and 2016 in our routine diagnostic laboratory for reduced susceptibility to carbapenems (CP) by using meropenem-containing media. Actively screened CP non-susceptible strains as well as 367 archived ESBL/AmpC-β-lactamase-producing *Enterobacteriaceae* were then tested for the presence of CP genes by PCRs. Among 21,569 isolates, OXA-48 could be identified as the sole carbapenemase type in 137 (0.64%) strains. The *bla*_OXA-48_ gene was located on an ∼60-kb IncL plasmid and sequence analysis revealed high similarity to reference plasmid pOXA-48a, which has been involved in the global spread of the *bla*_OXA-48_ gene in humans for many years. *Klebsiella pneumoniae* was the predominant OXA-48 producer (*n* = 86; 6.6% of all *K. pneumoniae* isolates), followed by *E. cloacae* (*n* = 28; 5.0%), *Klebsiella oxytoca* (*n* = 1; 0.3%), and *E. coli* (*n* = 22, 0.1%). OXA-48 was not found in livestock, but in dogs (120/3182; 3.8%), cats (13/792; 1.6%), guinea pig (1/43; 2.3%), rat (1/23; 4.3%), mouse (1/180; 0.6%), and one rabbit (1/144; 0.7%). Genotyping identified few major clones among the different enterobacteria species, including sequence types ST11 and ST15 for *K. pneumoniae*, ST1196 for *E. coli*, and ST506 and ST78 for *E. cloacae*, most of which were previously involved in the dissemination of multidrug-resistant strains in humans. The majority of OXA-48 isolates (*n* = 112) originated from a university veterinary clinic (UVC), while animals from further 16 veterinary institutions were positive. Clonal analyses suggested nosocomial events related to different species and STs in two veterinary clinics and horizontal transfer of the pOXA-48-like plasmid between bacterial species and animals. A systematic monitoring is urgently needed to assess the dissemination of CPE not only in livestock but also in companion animals and veterinary clinics.

## Introduction

According to the World Health Organization, carbapenems (CP) are intended to be restrictively used for the treatment of serious human diseases caused by multidrug resistant bacteria (Anonymous, 2017). Also as a response to the increased incidence of ESBL- and AmpC-producing *Enterobacteriaceae* the worldwide usage of CP has increased by approximately 40% in recent years (Liu et al., 2016; Loucif et al., 2016b), promoting the successful spread of carbapenemases in many parts of the world (Anonymous, 2017). Carbapenemases are a diverse group of β-lactamases that hydrolyze not only CP but also other beta-lactam antibiotics. Among the major carbapenemase types are the *K. pneumoniae* carbapenemases (KPC), the New Delhi Metalloprotease (NDM), and the class D β-lactamase OXA-48. OXA-48 was first detected in Turkey in 2001 before it spread through the Mediterranean countries to Western Europe (Poirel et al., 2012b; Van Boeckel et al., 2014). OXA-48-producing *Enterobacteriaceae* have been involved in hospital outbreaks in many parts of the world including Germany (Anonymous, 2016). The global dissemination of OXA-48 has been mainly attributed to a conjugative IncL/M plasmid of approximately 60 kb in size that harbors *bla*_OXA-48_ within Tn*1999* transposon structures (Poirel et al., 2012b; Carattoli et al., 2015; Pitout et al., 2015). In addition, clonal spread of certain OXA-48-producing strains, such as *K. pneumoniae* of ST11, ST15, and ST101, has been described (Voulgari et al., 2013; Branas et al., 2015; Jayol et al., 2016; Loucif et al., 2016b).

In recent years, the increasing number of reports about carbapenemases in *Enterobacteriaceae* in livestock and companion animals has raised a serious concern about the role of animals in the spread of CP-resistant bacteria and/or of plasmids that carry carbapenemase genes (Fischer et al., 2012; Shaheen et al., 2013; Schmiedel et al., 2014; Braun et al., 2016; Dolejska et al., 2016; Gonzalez-Torralba et al., 2016; Hamza et al., 2016; Liu et al., 2016; Loucif et al., 2016a; Yousfi et al., 2016, 2017; Melo et al., 2017; Pulss et al., 2017). In 2013, we described the emergence of OXA-48, which to that time was already highly disseminated in human patients, for the first time in isolates from dogs and cats in Germany (Stolle et al., 2013). Since then, further sporadic findings of OXA-48-like carbapenemases were reported from companion and livestock animals in different parts of the world (Schmiedel et al., 2014; Al Bayssari et al., 2015; Braun et al., 2016; Hamza et al., 2016; Liu et al., 2016; Yousfi et al., 2016, 2017; Melo et al., 2017; Pulss et al., 2017).

In the present study, we investigated the emergence of carbapenemase-producing *Enterobacteriaceae* (CPE) in companion animals and livestock. We describe the chronology of a long-term spread of OXA-48-producing *Enterobacteriaceae* in a veterinary teaching hospital for small animals in Germany and could additionally demonstrate that OXA-48-positive bacteria and/or OXA-48 plasmids have independently emerged in several veterinary clinics throughout Germany.

## Materials and Methods

### Bacterial Isolates, Screening for Carbapenem Resistance, and Detection of β-Lactamase Genes

From June 2012 to December 2016, 19,025 *E. coli*, 1607 *Klebsiella* spp., and 570 *Enterobacter* spp. isolates that were obtained in our veterinary microbiological laboratory from clinical and fecal samples of different animal species predominantly from Germany were tested for reduced susceptibility to CP using 1 ml MH broth containing a 10-μg meropenem disk (June 2012–March 2015) or MH agar supplemented with 0.5 μg/ml meropenem (April 2015–December 2016), respectively. Strains that showed growth in the screening media were further tested for the presence of carbapenemase genes *bla*_NDM-like_, *bla*_KPC-like_, *bla*_VIM-like_, and *bla*_OXA-48-like_ by PCR (Grobner et al., 2009; Pfeifer et al., 2011; Stolle et al., 2013). In case a carbapenemase gene was detected, the strains were additionally tested for ESBL genes (*bla*_CTX-M-like_, *bla*_SHV-like_, *bla*_TEM-like_, and *bla*_OXA-like_) and AmpC-β-lactamase genes (*bla*_CMY-like_, *bla*_DHA-like_, *bla*_ACC-like_, and *bla*_FOX-like_) using specific primers and conditions previously described (Grimm et al., 2004; Ewers et al., 2010; Chen et al., 2015; Yousfi et al., 2017). After amplification of beta-lactamase genes and carbapenemase genes, all positive amplicons were sequenced by a commercial sequencing company (LGC GmbH, Berlin, Germany).

Additional 367 archived ESBL/AmpC β-lactamase producing *Enterobacteriaceae* isolates, namely 247 *E. coli*, 70 *Klebsiella* spp., and 50 *Enterobacter* spp. that had been isolated from clinical specimens between June 2009 and May 2012 in Germany, were tested for the presence of the above mentioned carbapenemase genes by PCR (Grobner et al., 2009; Pfeifer et al., 2011; Stolle et al., 2013). The overall 21,569 *Enterobacteriaceae* isolates (19,272 *E. coli*, 1,307 *K. pneumoniae*, 365 *K. oxytoca*, 5 *Klebsiella* spp., 438 *E. cloacae*, 81 *E. amnigenus*, 49 *E. aerogenes*, and 52 isolates belonging to other *Enterobacter* spp.) were received from pigs (*n* = 10,899), cattle (*n* = 1,889), small ruminants (*n* = 246), birds (*n* = 132), dogs (*n* = 3,775), cats (*n* = 932), horses (*n* = 2,573), pet animals and rodents (*n* = 428), zoo and wildlife animals (*n* = 644), and from other or unknown animal species (*n* = 51).

Isolates were obtained from the feces and gastrointestinal tract (*n* = 14,155), genital tract (*n* = 2,211), respiratory tract (*n* = 1,287), organs (*n* = 1,077), urinary tract (*n* = 907), skin/hair (*n* = 433), wounds (*n* = 404), ear (*n* = 163), abdominal cavity/ascites (*n* = 113), abscess/fistula (*n* = 139), milk/udder (*n* = 83), eye (*n* = 53), central venous catheter (*n* = 52), blood (*n* = 22), and from diverse other locations (*n* = 470).

The majority of fecal samples (75%) were from pigs and were sent to our diagnostic laboratory for molecular typing of virulence-associated genes that are predictive for *E. coli* pathovars implicated in swine diarrhea and edema disease. In case different virulence gene profiles were determined, a maximum of three isolates per sample was delivered to the present study. Porcine isolates previously published (Pulss et al., 2017) were not included in this study.

As the study did not follow a specific sampling schedule but included isolates obtained along with an in-house veterinary microbiological diagnostic service and also archived strains, the number of investigated samples per year differed (2009, 43 isolates; 2010, 102 isolates; 2011, 146 isolates; 2012, 1730 isolates; 2013, 3675 isolates; 2014, 3573 isolates; 2015, 6614 isolates; 2016, 5686 isolates).

### Antimicrobial Susceptibility Testing

Antimicrobial susceptibility testing was performed for all isolates carrying a carbapenemase gene using the VITEK^®^ 2 system (AST-card GN38; bioMérieux, Nürtingen, Germany). Minimum inhibitory concentrations (MICs) were interpreted according to breakpoints set by the Clinical and Laboratory Standards Institute (CLSI) for human and veterinary (in case of enrofloxacin, marbofloxacin, ceftiofur, and gentamicin) *Enterobacteriaceae* isolates (Clinical and Laboratory Standards Institute [CLSI], 2013, 2017). Polymyxin B MICs were interpreted according to manufacturer analogous to Colistin breakpoints given by the European Committee on Antimicrobial Susceptibility Testing (EUCAST). Colistin MICs were further evaluated using microdilution as recommended by the EUCAST and the CLSI^1^.

### Multilocus Sequence Typing (MLST) and Clonal Analysis by Pulsed-Field Gel Electrophoresis

MLST was performed using seven housekeeping genes each of *K. pneumoniae* and *K. oxytoca* (*gapA, infB, mdh, pgi, phoE, rpoB*, and *tonB*), *E. cloacae* (*dnaA, fusA, gyrB, leuS, pyrG, rplB*, and *rpoB*), and *E. coli* (*adk, fumC, gyrB, icd, mdh, purA*, and *recA*). Details about the MLST schemes, including the primers, PCR conditions, and methods for allelic and sequence type assignment are available at MLST databases of the Institute Pasteur for *K. pneumoniae*^2^ and *E. coli*^3^ as well as at the PubMLST database in case of *K. oxytoca*^4^ and *E. cloacae*^5^.

Macrorestriction analysis and pulsed-field gel electrophoresis (PFGE) was performed as described previously (Ewers et al., 2010). Profiles were compared using BioNumerics (Version 6.6, Applied Maths, Belgium) and cluster analysis of Dice similarity indices were performed based on UPGMA.

### Genomic Location of Carbapenemase Genes

To determine the genomic location of carbapenemase genes, plasmid DNA, and I-CeuI digested whole-cell DNA were separated by agarose and PFGE, respectively, and analyzed by Southern blot hybridization. DNA probes were prepared using the PCR Dig Probe Synthesis Kit (Boehringer Mannheim GmbH, Germany) and consisted of a 1,486 bp PCR fragment specific for 16S rRNA genes (primers SK16R/SK16F), and an internal PCR fragment specific for the *bla*_OXA-48-like_ gene (743 bp; primers OXA-48A/B), respectively (Stolle et al., 2013). Detection of hybridized DNA molecules was done with the DIG Luminescent Detection Kit (Boehringer Mannheim GmbH, Mannheim, Germany).

### Transfer of Carbapenemase Genes by Conjugation

To test whether carbapenemase genes were transferable, conjugation was performed by the filter mating method at 37°C on 48 OXA-48 positive isolates representing different species and multilocus sequence types (**Supplementary Table S1**) using plasmid-free sodium azide resistant *E. coli* J53 (J53 Azi^R^) as recipient. Transconjugants were selected on Mueller Hinton agar plates supplemented with 100 mg/L sodium azide and 0.5 mg/L meropenem (Sigma-Aldrich, Germany). To confirm transfer of carbapenemase genes, antimicrobial susceptibility testing and PCR was performed as described. PCR-based replicon typing (PBRT) of the main plasmid incompatibility groups reported in *Enterobacteriaceae* was performed by using a commercially available PBRT kit (DIATHEVA, Cartoceto, Italy) (Carattoli et al., 2005).

### Plasmid Profile and Plasmid Sequence Analysis

Plasmid profiles and estimated sizes were determined using S1 nuclease restriction of genomic DNA and subsequent PFGE analysis (Stolle et al., 2013). For sequencing of selected OXA-48 plasmids, the plasmid DNA was harvested from *E. coli* J53 transconjugants using a QIAfilter Plasmid Midi Kit (Qiagen GmbH, Hilden, Germany). Plasmid preparation and sequencing (using illumina MiSeq V2) was done by LGC Genomics GmbH, Berlin. Sequence data were assembled *de novo* using SPAdes Genome Assembler V. 3.8^6^ and sequences were annotated using the Rapid Annotation using Subsystem Technology (RAST) server^7^. PCR mapping was performed to determine the correct order of plasmid contigs and sequence assemblies using primers listed in **Supplementary Table S1**. The presence of known plasmid contexts within the assemblies that were related to previously sequenced ones, such as pKPoxa-48N1 (GenBank accession number NC_021488) and Kp11978-pOXA-48 (NC_019154) was further identified by a BLAST search^8^ of publicly available plasmid sequences using Geneious version 7.2.1^9^ (Mairi et al., 2017). A circular visualization of *bla*_OXA-48_ containing plasmids was performed using BRIG^10^.

### Nucleotide Sequence Accession Numbers

The nucleotide sequences of OXA-48 plasmid contigs were deposited at GenBank under the accession numbers MH213677-MH213682 (pIHIT22059), MH213683-MH213688 (pIHIT22060), MH213689-MH213694 (pIHIT23114), MH213695-MH213699 (pIHIT25327), MH213700-MH213706 (pIHIT25329), MH213707-MH213711 (pIHIT25661), MH213712-MH213717 (pIHIT27939), MH213718-MH213722 (pIHIT28629), and MH213721-MH213730 (pIHIT29873).

## Results

### Distribution of Carbapenemase Genes

We identified 137 (0.64%) isolates that carried a *bla*_OXA-48-like_ gene which was determined as *bla*_OXA-48_ in all cases. Included are eight strains (5 *K. pneumoniae* and 3 *E. coli* strains) that have been published previously (Stolle et al., 2013). The percentage of isolates carrying *bla*_OXA-48_ was 14.98% for the 367 archived ESBL/AmpC-β-lactamase producing strains and 0.43% for the isolates obtained by active screening. None of the 21,569 *Enterobacteriaceae* isolates harbored any of the other carbapenemase genes tested.

OXA-48 positive strains were exclusively found in isolates from non-livestock animals, particularly from dogs (120/3775; 3.2%) and cats (13/932; 1.4%). Single *K. pneumoniae* strains were recovered from a guinea pig (1/43; 2.3%), a rat (1/23; 4.3%), a mouse (1/180; 0.6%) and a rabbit (1/144; 0.7%). *K. pneumoniae* was the predominant carbapenemase producer in numbers (*n* = 86), followed by *E. cloacae* (*n* = 28), *E. coli* (*n* = 22), and *K. oxytoca* (*n* = 1). Also with respect to the within-species frequency, *K. pneumoniae* was the most frequent OXA-48 producer (6.6%), followed by *E. cloacae* (5.0%), *K. oxytoca* (0.3%), and *E. coli* (0.1%). Referring to dogs and cats, 23.4% (77/329 isolates) and 11.4% (5/44) of the *K. pneumoniae* isolates were OXA-48 positive, respectively. The percentage of positive isolates from dogs and cats was 15.4% (21/136) and 11.3% (7/62) for *E. cloacae* and 0.7% (21/3182) and 0.1% (1/792) for *E. coli*.

Animals carrying OXA-48 positive isolates originated from different veterinary clinics and/or households in Germany (**Supplementary Document S1**). The majority of OXA-48 producers (*n* = 112; 81.8%) originated from animals that were hospitalized in a university veterinary clinic (UVC). Additional five strains were obtained from organ samples received after necropsy of animals in the Institute of Veterinary Pathology (IVP) located on the same campus (distance ca. 0.3 km). The remaining strains were isolated from animals that had been admitted to 15 different veterinary practices and clinics (termed clinic-1 to clinic-15) located in different geographic regions in Germany (distances between 13 km and 450 km; *n* = 20 isolates). All non-UVC and non-IVP institutions are referred to as “external clinics”, the corresponding strains as “external strains” in the manuscript. Among the 20 external strains were 13 singletons, i.e., isolated from animals in 13 different clinics, while OXA-48 producers could be repeatedly isolated from two institutions, namely clinic-3 (5 isolates) and clinic-10 (2 isolates) (**Table 1**).

**Table 1:** Distribution of OXA-producing *Enterobacteriaceae* isolates according to bacterial species, date and source of isolation.

Veterinary institutions	Species	No. of isolates	Date of isolation	Isolation source^∗^	Pure culture	Host^∗^	Epidemiologic link to UVC
UVC	*K. pneumoniae*	79	08/2009-08/2016	urine (21), wound (20), CVK (12), abdominal cavity (6), skin (4), blood (3), others (13)	yes (24), no (55)	dog (75), cat (4)	
	*K. oxytoca*	1	11/2012	urine	no	dog	
	*E. cloacae*	14	07/2010-08/2015	CVK (6), urine (2), tracheal tube (2), others (4)			
	*E. coli*	18	03/2012-10/2016	urine (4), wound (4), CVK (4), BAL (2), others (6)	yes (5), no (13)	dog (17), cat (1)	
**External clinics & IVP**							
IVP	*K. pneumoniae*	4	08/2010-10/2010	liver (2), nose, lung	no	guinea pig, rat, rabbit, mouse	no
	*E. cloacae*	1	06/2012	liver	no	dog	yes
Clinic 1	*K. pneumoniae*	1	10/2010	nose	no	dog	no
Clinic 2	*E. coli*	1	10/2012	urine	yes	dog	yes
Clinic 3	*E. cloacae*	4	03/2013-09/2016	axillary swab, skin, urine, nose	yes (1), no (3)	dog (2), cat (2)	no
	*E. coli*	1	04/2014	throat	no	dog	no
Clinic 4	*E. cloacae*	1	01/2013	trachea	no	dog	no
Clinic 5	*E. cloacae*	1	05/2014	urine	unknown	dog	no
Clinic 6	*E. cloacae*	1	07/2014	tissue	no	cat	no
Clinic 7	*K. pneumoniae*	1	11/2014	skin	no	dog	yes
Clinic 8	*E. cloacae*	1	12/2014	wound	no	cat	no
Clinic 9	*E. cloacae*	1	12/2014	trachea	no	dog	no
Clinic 10	*E. cloacae*	2	06/2015, 08/2015	ascites, bulla	yes (1), no (1)	dog (2)	no
Clinic 11	*E. cloacae*	1	08/2015	wound	no	dog	no
Clinic 12	*E. coli*	1	12/2016	urine	no	dog	no
Clinic 13	*E. cloacae*	1	12/2016	bile	no	cat	no
Clinic 14	*E. cloacae*	1	10/2016	urine	unknown	dog	no
Clinic 15	*K. pneumoniae*	1	10/2016	urine	unknown	cat	no

*^∗^The number of isolates is given in brackets; UVC, University Clinic; IVP, Institute of Veterinary Pathology; CVK, central venous catheter; BAL, broncho-alveolar lavage.*

According to the year of isolation, a minimum of two strains (year 2009) and a maximum of 29 strains (year 2012) were detected. The relative proportion of OXA-48 positive strains from dogs and cats according to study year differs from 11.1% to 37.5% in the retrospective screening period (2009 to May 2012), where preselected ESBL/AmpC-β-lactamase isolates were investigated, and from 1.0% to 3.7% in the active screening period, where isolates had not been preselected for a certain resistance phenotype (**Supplementary Figure S1**). The four OXA-48 positive strains from animals other than dogs or cats were obtained in the year 2010 and were all isolated in the IVP.

### Distribution of Other β-Lactamase Genes Among OXA-48 Positive Isolates

Sixty-one isolates (44.5%) carried the ESBL gene *bla*_CTX-M-15_ and one *K. pneumoniae* isolate was positive for *bla*_CTX-M-27_ (**Supplementary Figures S2A–C**) Fifty-six (65.5%) *K. pneumoniae* isolates carried *bla*_CTX-M-15_ while this was the case for 18.2% and 3.6% of the *E. coli* and *E. cloacae* isolates, respectively. The single *K. oxytoca* isolate was negative for any of the additionally tested β-lactamase genes. AmpC β-lactamase genes *bla*_DHA-1_ and *bla*_CMY-2_ were identified in 20 (14.6%) and 10 (7.3%) of the 137 OXA-48 positive isolates, respectively. The DHA-1 gene was found in *K. pneumoniae* (20.9%) and in *E. cloacae* isolates (9.1%), while the CMY-2 gene was almost exclusively determined in *E. coli* isolates (8/22; 36.4%) and in only two *K. pneumoniae* isolates (2/86; 2.3%). Furthermore, 86 (62.8%) of all OXA-48 producing isolates were positive for *bla*_TEM-1_ and 76 (55.5%) isolates for *bla*_OXA-1_, while 104 (75.9%) carried *bla*_SHV-like_ genes, including *bla*_SHV-1_ (2/137; 1.5%), *bla*_SHV-2_ (1.5%), *bla*_SHV-11_ (13.1%), *bla*_SHV-12_ (11.7%), *bla*_SHV-27_ (0.7%), and *bla*_SHV-28_ (47.4%) as shown in **Supplementary Figures S2A–C**. Among the 86 *K. pneumoniae* isolates, SHV-28 was the predominant SHV-type (75.6%) while only few strains harbored SHV-2, SHV-11, and SHV-27. The SHV-12 enzymes were only detected among *E. cloacae* and *E. coli* isolates (31.1% and 31.8% *bla*_SHV-12_ positive, respectively).

### Antimicrobial Susceptibility

Forty-six (33.6%) of the 137 OXA-48 positive *Enterobacteriaceae* isolates were resistant to imipenem (≥4 mg/L). The remaining isolates revealed MICs of either 2 mg/L (49 isolates, 35.8%) or ≤1 mg/L (42 isolates, 30.7%). *E. cloacae* was the species with the highest frequency of imipenem resistance (78.6% of 28 isolates), followed by *K. pneumoniae* (29.1%), whereas none of the *bla*_OXA-48_ positive *E. coli* isolates was resistant to imipenem. Sixty-eight (49.6%) isolates had an ESBL phenotype, which was mainly due to the presence of CTX-M enzymes. All strains were resistant to ampicillin, piperacillin, and the amoxicillin–clavulanate combination. The majority of strains were also resistant to the tested fluoroquinolones with percentages per species ranging from 90.0 to 100%. (**Table 2**). Moreover, different rates of aminoglycoside resistances were determined, with percentages ranging between 0 for amikacin, 49.6% for tobramycin, and 52.9% for gentamicin (**Table 2**).

**Table 2:** Antimicrobial susceptibility of 137 OXA-48-positive *Enterobacteriaceae* strains isolated from clinical samples of companion animals.

	*K. pneumoniae/ K. oxytoca*	*E. cloacae*	*E. coli*

	Resistance rate [*n* (%)]		
Ampicillin^1^	87 (100)	28 (100)	22 (100)
Amoxicillin-clavulanic acid^1^	87 (100)	28 (100)	22 (100)
Piperacillin^1^	87 (100)	28 (100)	22 (100)
Cefalexine^1^	76 (87.4)	28 (100)	8 (36.4)
Cefpodoxime^1^	72 (82.8)	20 (71.4)	14 (63.6)
Ceftiofur^2^	77 (88.5)	27 (96.4)	21 (95.5)
Cefpirome^1^	56 (64.4)	6 (21.4)	4 (18.2)
Imipenem^1^	26 (29.9)	22 (78.6)	0
Amikacin^1^	0	0	0
Gentamicin^2^	44 (50.6)	14 (50.0)	12 (54.5)
Tobramycin^1^	46 (52.9)	15 (53.6)	7 (31.8)
Enrofloxacin^2^	86 (98.9)	27 (96.4)	20 (90.9)
Marbofloxacin^2^	86 (98.9)	27 (96.4)	20 (90.9)
Tetracycline^1^	43 (49.4)	21 (75.0)	20 (90.9)
Nitrofurantoin^1^	26 (29.9)	6 (21.4)	0
Trimethoprim-sulfamethoxazole^1^	50 (57.5)	27 (96.4)	18 (81.8)
Chloramphenicol^1^	22 (25.3)	13 (46.4)	17 (77.3)
Polymyxin B^1^	0	0	0


*^*1*^Interpreted according to CLSI, Performance Standards for Antimicrobial Susceptibility Testing, 27th ed. CLSI supplement M100, 2017. ^*2*^Interpreted according to CLSI, Performance Standards for Antimicrobial Disk and Dilution Susceptibility Tests for Bacteria Isolated from Animals, 3rd ed. CLSI supplement VET01S, 2015.*

### Multilocus Sequence Types, Clonal Relatedness, and Plasmid Profiles of OXA-48 Producing Bacteria

**FIGURE 1:**
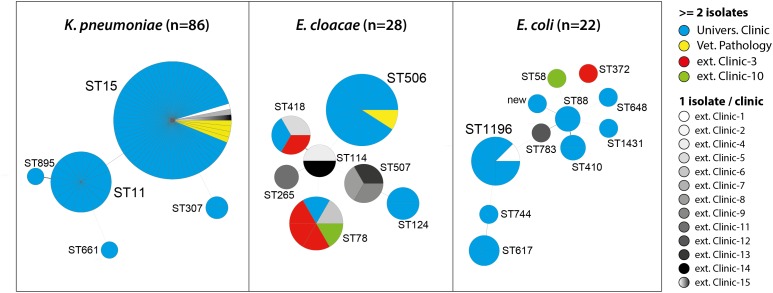
Multilocus sequence types (STs) of OXA-48 positive *Enterobacteriaceae* from clinical samples of companion animals with respect to strain origin.

**FIGURE 2:**
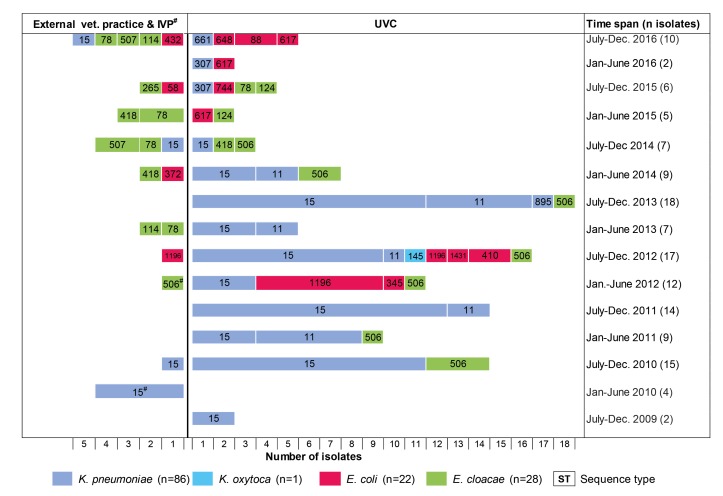
Chronology of appearance of OXA-48-producing *Enterobacteriaceae* in a University Veterinary Clinic (UVC) and the Institute of Veterinary Pathology (IVP) located on the same campus as well as in 15 veterinary external clinics outside the campus.

The 86 OXA-48 positive *K. pneumoniae* isolates were predominantly assigned to sequence types ST15 (75.6%) and ST11 (19.8%) (**Figure 1**). Other sequence types determined were ST307 (*n* = 2), ST661 (*n* = 1), and ST895 (*n* = 1), which is a single locus variant (SLV) of ST11. ST11 and ST15 strains were mainly observed in the UVC (87.2%) and the epidemiologically linked IVP (4.7%) and were frequently found between July 2009 and June 2014, before they almost disappeared after that time period (**Figure 2**). Major STs among the 28 *E. cloacae* strains were ST506 (39.3%), which was found in the UVC and the IVP but in none of the external clinics, and ST78 (21.4%). Further STs identified were ST114 (*n* = 2) and its SLV ST418 (*n* = 3) as well as ST124 (*n* = 2), ST265 (*n* = 1), and ST507 (*n* = 3). In contrast to the other species, *E. cloacae* strains were highly dispersed with regard to clinic background, as they were found in animals from 10 external veterinary clinics.

With the exception of ST1196, which was the predominant ST (36.4%) among the 22 *E. coli* strains and appeared in 2012 in the UVC and the IVP (**Figure 2**), the remaining strains revealed a high diversity and were distributed among 10 different STs, including ST648 and ST410, which have previously been described as emergent ESBL producing clones in various hosts, including humans (Ewers et al., 2014a; Poirel et al., 2014; Fernandez et al., 2015). Also, our ST648 isolate carried a CTX-M-15 β-lactamase, while the two ST410 isolates were no ESBL producers.

PFGE analysis grouped isolates basically according to their multilocus sequence types (**Supplementary Figures S2A–C**). ST15- *K. pneumoniae* isolates clustered together based on similarity values of 86.0% to 100% (*Kp*-pulsotype D). In case of *Kp*-ST11 isolates, the similarity values ranged from 92.0% to 100% (*Kp*-pulsotype C), while isolates of ST307 (*Kp*-pulsotype A), ST661 (*Kp*-pulsotype E), and ST895 (*Kp*-pulsotype B) clustered separately from each other and in addition from ST15 and ST11 (< 85% similarity) (**Supplementary Figure S2A**). A similar clustering based on assignment to STs was observed for *E. cloacae* and *E. coli* isolates. In detail, *E. coli* isolates were grouped into *Ec*-pulsotypes A-J, and except for ST617 and ST88 isolates, which clustered at a similarity value of 85.7%, all other STs belonged to distinct pulsotypes (**Supplementary Figure S2B**). The 28 *E. cloacae* isolates were separated into *Ecl*-pulsotypes A-F (**Supplementary Figure S2C**), which perfectly reflects their ST assignment.

We identified 11 plasmid profiles among the 22 *E. coli*, 19 profiles among *E. cloacae*, and 21 profiles among *K. pneumoniae* isolates (**Supplementary Figures S2A–C**). This indicates an overall high diversity of plasmid content and suggests that strains may frequently lose and gain plasmids in their different habitats. However, we also found several isolates that revealed not only identical STs, but also high relatedness in terms of PFGE and/or plasmid patterns. This was for example the case for different subgroups of ST11- and ST15-*K. pneumoniae* strains that presented identical plasmid profiles and highly related macrorestriction profiles although originating from different time points, clinics, and host species (**Supplementary Figure S2A**). The repeated isolation of pulsotype-identical ST15-*K. pneumoniae* isolates from dog-86 in the UVC (where the dog underwent surgery) in October 2014 and in clinic-7 (aftercare-treatment) and the possibility in November 2014, indicates that animals may transfer OXA-48 producing Enterobacteriaceae between different clinics, eventually by contaminating the clinical environment. Regarding *E. coli*, one ST1196 strain isolated from dog-49 in clinic-2 in 2012 revealed >98% similarity in its pulsotype and an identical plasmid profile as compared to six strains of the same ST, that were isolated in the UVC from other dogs and cats a few months earlier (**Supplementary Figure S2C**).

For *E. cloacae*, we found identical plasmid patterns among 7 out of 11 ST506 strains, all obtained from the UVC over a time period of about 4 years (**Supplementary Figure S2B**). Likewise, identical PFGE and plasmid patterns suggested clonality for three ST507-*E. cloacae* strains isolated from two cats (cat-6, cat-12) and one dog (dog-87) in three different clinics (clinic-8, -9, and -13) in the years 2014 and 2016. Finally, ST78-*E. cloacae* isolates, obtained from the UVC (dog-94), clinic-3 (dog-60 and dog-89), and clinic-6 (cat-5) were highly similar (≥92.1%) based on their PFGE profiles. Here, however, apart from the regular presence of the ∼60-kb OXA-48 plasmid, plasmid profiles showed only partial overlap which may indicate an ongoing exchange of non-OXA-48 plasmids and/or a different background of the strains.

### Genomic Location of OXA-48 Plasmids and Conjugation Experiments

S1-nuclease restriction analysis and subsequent hybridization analysis revealed the presence of an approximately 60 kb-plasmid and the localization of the *bla*_OXA-48_ gene on this plasmid for the 137 OXA-48 positive isolates (data not shown). All 48 isolates selected for transconjugation assays based on their assignment to different enterobacterial species, STs, year of isolation, and MIC values for imipenem, successfully transferred the *bla*_OXA-48_ gene to the recipient *E. coli* J53. Plasmid profile analysis and replicon typing revealed the presence of a ∼60-kb IncL plasmid in all transconjugants (TCs). All TCs exhibited an increase of four- to fivefold in MICs to ampicillin and piperacillin compared to the recipient strain. MICs to amoxicillin/clavulanate increased by two- to fourfold, those to the cephalosporins, cefpodoxim, ceftiofur, and cefpirom were either the same as in the recipient strain or showed an increase by two-to fivefold (**Supplementary Table S1**). In the majority of the TCs (91.7%), the transfer of the OXA-48-plasmid resulted in an increased MIC to imipenem in the recipient strain. In detail, MICs of 2 mg/L, 4 mg/L, and 8 mg/L were determined in 23, 19, and two of the TCs, respectively.

### Sequence Analysis of OXA-48 Plasmids

**FIGURE 3:**
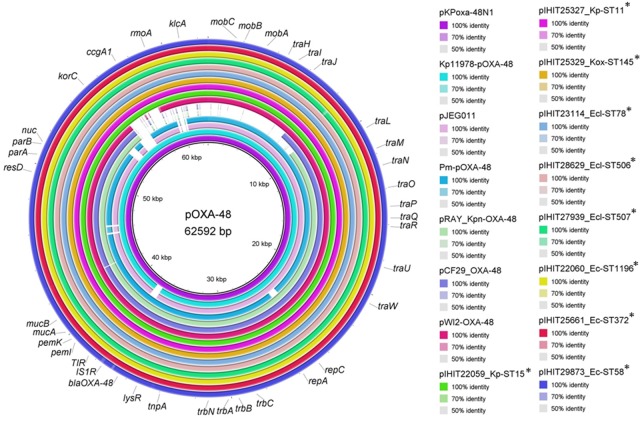
Sequence comparison of plasmid pKPoxa-48N1 and other published OXA-48 plasmids with plasmid sequence contigs of OXA-48 plasmids from animal *Enterobacteriaceae.* Plasmids (GenBank accession number, species, host, country, year of isolation) used for comparison: pKPoxa-48N1 (NC_021488; *K. pneumoniae*, human, France, 2010) (Berger et al., 2013); Kp11978-pOXA-48a (NC_019154, *K. pneumoniae*, human, Turkey, 2001) (Poirel et al., 2012b); pJEG011-Kp002 (KC354801.1, *K. pneumoniae*, human, Australia, 2010) (Espedido et al., 2013); pRAY_Kpn-OXA-48 (KX524525, *K. pneumoniae*, human, Italy, 2013) (Touati et al., 2017), pWI2-OXA-48 (LT838202, *E. coli*, human) [direct submission to NCBI database], pCF29_OXA-48 (LN864820; *Citrobacter freundii*, Lebanon) (direct submission to NCBI database), pOXA-48-Pm (KP025948, *Proteus mirabilis*, human, Palestine, 2012) (Chen et al., 2015).

We comparatively analyzed selected *bla*_OXA-48_ encoding IncL plasmid sequences from our study with published sequences of human OXA-48 plasmids that have previously been described from various bacterial species (**Figure 3**) (Poirel et al., 2012b; Berger et al., 2013; Espedido et al., 2013; Chen et al., 2015; Touati et al., 2017). Like in pKPoxa-48N1, *bla*_OXA-48_ was part of the composite transposon Tn*1999.2*, that was first described in the context of the spread of OXA-48-producing *K. pneumoniae* isolates in Turkey (Carrer et al., 2008; Berger et al., 2013). In contrast to Tn*1999.1*, which was identified in the prototype plasmid pOXA-48a in *K. pneumoniae* strain 11978 (Poirel et al., 2012b), the Tn*1999.2* isoform contains an IS*1R* element inserted into the IS*1999* element located upstream of *bla*_OXA-48_. In addition, we could detect almost the complete sequence and gene organization of pKPoxa-48N1 in the plasmid contigs of our isolates with an overall coverage of 98% and sequence identity of 99%. Irrespective of the enterobacterial isolate, host, and clinic origin, the animal OXA-48 plasmids revealed almost sequence identity. **Figure 3** provides a circular view of OXA-48 plasmids from nine animal isolates representing different species and STs and obtained from the UVC and from external clinics as compared with the reference plasmids. Plasmid mapping of the remaining 39 *bla*OXA-48 carrying plasmids of transconjugants by 22 PCRs spanning almost the entire plasmid sequence (see **Supplementary Table S2**) revealed that all 48 plasmids showed a positive result with the expected amplicon sizes, as referred to the reference plasmid pKPoxa-48N1.

## Discussion

Our results suggest that OXA-48 type carbapenemases are on their way to successfully entering the companion animal population and/or veterinary clinics in Germany. These findings add up to a growing number of reports about the emergence of OXA-48-CPE in cats, dogs, horses, and pet birds in Germany (Stolle et al., 2013; Schmiedel et al., 2014), Algeria (Yousfi et al., 2016; Yousfi et al., 2017), France (Melo et al., 2017), and the United States (Liu et al., 2016). In the present study, guinea pig, rat, mouse, and rabbit could be identified as an additional source of CPE, suggesting a broader host spectrum of such bacteria than previously known.

We observed a frequency of 0.43% OXA-48-producers among 21,202 Enterobacteriaceae that were not preselected for any antimicrobial resistance. This basically reflects numbers from previous studies, where 0.26% and 2.3% OXA-producing bacteria have been determined among 383 non-preselected isolates from healthy cats and dogs in France (Melo et al., 2017) and 533 rectal swabs obtained from healthy and diseased pets in different cities in Algeria (Yousfi et al., 2016), respectively. Not unexpected, the frequency of OXA-48-producers was considerably higher among our set of 367 archived ESBL-/AmpC-β-lactamase producing isolates (14.98%). Similarly, Liu et al. (2016) determined OXA-48 in 19.1% of 68 ESBL-producing *E. coli* isolates from diseased dogs and cats in the United States, supporting the frequent multidrug-resistant (MDR) phenotype of CPE (Liu et al., 2016). Indeed, almost 50% of our OXA-48-producing isolates showed an ESBL phenotype and high rates of resistances to other antimicrobials, including fluoroquinolones, aminoglycosides, penicillins, cephalosporins, and tetracycline, probably due to antibiotic selective pressure. Data regarding previous antibiotic treatment were only partially available, but at least 13 (9.5%) of the animals carrying OXA-48-producing bacteria had received cephalosporins (2nd or 3rd generation), while 27.7%, 40.1%, and 60.6% had been treated with aminopenicillins, fluoroquinolones, and amoxicillin-clavulanic acid, respectively, either prior to their admission to a clinic or during their stay. It has been documented, that not only CP, which are not registered for use in veterinary medicine, but also other classes of antimicrobials, including cephalosporins, fluoroquinolones, aminopenicillins, or penicillin-β-lactamase inhibitor combinations might co-select for carbapenemase genes and contribute significantly to the development of resistance to CP (Poirel et al., 2012a; Jiao et al., 2015).

Our previous work was the first to suggest a nosocomial dissemination of OXA-48 in a veterinary clinical setting (Stolle et al., 2013). Here, five OXA-48-producing *K. pneumoniae* (ST15) and three *E. coli* (ST1196 and ST1431) recovered from six diseased dogs, admitted to the UVC in 2012, were described. The present study extends these data considerably with respect to the investigation period (2009–2016), number of strains, and involved veterinary clinics, providing insight into the chronology and clonal diversity of OXA-48 strains. The earliest OXA-48 producer was a ST15-CTX-M-15 *K. pneumoniae* strain isolated from a dog in the UVC in 2009. Nearly one year later, OXA-48-producing *E. cloacae* strains (ST506) appeared in the same clinic before we could identify an *E. coli* isolate (ST1196-SHV-12) carrying an OXA-48 gene for the first time in the year 2012. The second dominant *K. pneumoniae* clone (ST11-DHA-1) appeared nearly two years after the detection of the ST15 clonal group. Thus, *K. pneumoniae* ST15 seems to represent the starting point for the spread of OXA-48 in the UVC, although with the limitation that data from earlier strains were not available. In human medicine, epidemic clones of OXA-48-*K. pneumoniae* are often initially recovered prior to other OXA-48-producing *Enterobacteriaceae* isolates, and the horizontal transfer of OXA-48 plasmids from *K. pneumoniae* to *E. coli* within the gut of patients has been demonstrated (Goettig et al., 2015; Mairi et al., 2017). Also consistent with what is known for humans, *K. pneumoniae* was the most frequent OXA-48 producer in our sample material, probably reflecting the ability of this species to adapt to the hospital and veterinary clinical environment (Pitout et al., 2015; Anonymous, 2016). The sudden drop of ST11- and ST15-*K. pneumoniae* in the UVC in the second half-year of 2014 may be due to the implementation of improved cleaning and disinfection measures along with an increased hygiene control (personal communication).

The epidemic ST15-CTX-M-15 *K. pneumoniae* clone has recently been reported in the context of veterinary clinic-acquired infections in cats and dogs in France and Germany (Haenni et al., 2012; Ewers et al., 2014b), while ST11-*K. pneumoniae* carrying ESBL or AmpC β-lactamase genes were identified in companion animals in Spain and Italy (Hidalgo et al., 2013; Donati et al., 2014). Interestingly, ST11 and ST15 are also dominant among human OXA-48-producing *K. pneumoniae* isolates. Here, ST15 represents a globally disseminated clone carrying ESBL and carbapenemases, particularly in Europe and also in Germany (Kaase et al., 2016; Mairi et al., 2017). ST11 has been involved in outbreaks in Spain and Tunisia and is now found throughout Europe and other parts of the world (Mairi et al., 2017). Consistent with the frequent carriage of CTX-M-15 in human ST15 strains, >80% of the animal OXA-48-ST15 isolates co-produced this ESBL enzyme, whereas ST11 strains regularly co-expressed AmpC β-lactamase DHA-1. Apart from ST15 and ST11, ST307 isolated from a nasal and a wound swab from two dogs admitted to the UVC was recently identified in the feces of six patients in a hospital in Morocco. Like our strains, the human ST307 *K. pneumoniae* co-expressed the CTX-M-15 ESBL, again revealing similarities between human and animal strains (Girlich et al., 2014).

Consistent with previous reports from humans, animal *E. coli* isolates with OXA-48 were mostly polyclonal, with 11 different STs identified among 22 strains. Nevertheless, the repeated isolation of ST1196 from six animals in the UVC between March and October 2012 also suggested clonal dissemination. Identical plasmid patterns and co-expression of SHV-12 and CMY-2 was observed in all but one of the ST1196 strains, further supporting the clonal nature of the strains. This clone was also isolated from a dog (dog-49) in external clinic-2 about 2 weeks after a ST15-*K. pneumoniae* had been isolated from the same dog while he had stayed in the UVC due surgical treatment of a portosystemic shunt. This suggests an *in vivo* transfer of the OXA-48 plasmid between different bacterial species, like previously shown for humans and demonstrated for other animals in the present study (Goettig et al., 2015). In dog-42, three different OXA-48 strains, namely ST15-*K. pneumoniae*, ST1196-*E. coli*, and ST345-*E. coli* were isolated from a wound. Dog-95 carried an ST744-*E. coli* and an ST307-*K. pneumoniae* in one sample from the respiratory tract taken five days after hospitalization in the UVC due to severe aspergillosis. Both STs occurred for the first time in the UVC, suggesting an introduction of this strain and the OXA-48 plasmid by dog-95 or a transfer of the OXA-48 plasmid from one of the dominant clones circulating in the clinic to newly entered strains.

Among the OXA-48 producing *E. coli* we could identify STs which have previously been isolated from humans in different geographic areas (Mairi et al., 2017). A clonal distribution of ST648 occurred in Spain, the United Kingdom, and the United States; ST58 and ST410 were recorded in Spain, and ST1431 exhibited a clonal spread in Spain and Israel. Regarding ST1196, strains carrying the VIM-1 carbapenemase have recently been reported from human patients in Germany, while we are not aware about the emergence of OXA-48 in this sequence type (Kaase et al., 2015). ST410 was recently referred to as a successful clone involved in the spread of ESBL genes, and genetic similarities were identified between human and animal strains (Poirel et al., 2014). Similarly, ST648 is reported increasingly as an emerging resistance-associated genotype with extraintestinal virulence potential in humans and animals and its zoonotic potential has been indicated in previous studies (Pitout, 2012; Ewers et al., 2014a). Besides from our findings, ST648 was one of the six sequence types Liu et al. (2016) previously observed among 13 OXA-48 producing *E. coli* from diseased dogs in the United States. Another overlap in *E. coli* STs was revealed only recently, as Melo et al. (2017) identified an OXA-48-producing ST372 in a French dog, representing the first CPE in animals in that country .

*E. cloacae* is an opportunistic pathogen frequently involved in nosocomial infections and a number of studies have demonstrated the emergence of OXA-48-producing strains in hospitals worldwide (Poirel et al., 2012a; Potron et al., 2013; Fernandez et al., 2015; Findlay et al., 2017). It has been sporadically reported for companion animals as well (Schmiedel et al., 2014; Yousfi et al., 2017). MLST distinguished seven different STs among our 28 isolates with ST506 (39.3%), ST78 (21.4%), and ST114 together with its SLV ST418 (17.9%) being the predominant genotypes. Of note, ST114 and ST78 were among the major lineages described for MDR *E. cloacae* and have been identified as potential high-risk international clones in Europe (Girlich et al., 2015; Izdebski et al., 2015). Also other STs identified in the present study have been involved in the clonal transmission of MDR isolates, such as ST124 in a Chinese Tertiary Hospital (Cao et al., 2017). In contrast, ST506 represented a novel type, which was completely unrelated to known STs. This clone repeatedly occurred in the UVC and the IVP, located on the same campus, while it was not found in any external clinic. Notably, six ST506 strains isolated in different years, i.e., 2010, 2012, and 2014 revealed highly similar PFGE types and all possessed SHV-12 and TEM-1, indicating maintenance of the strains in the UVC for several years or repeated entry. Only recently, Yousfi et al. (2017) suggested that clonally related OXA-48-ST527-*E. cloacae* strains might have circulated among healthy dogs at different dates and geographical areas in Algeria. In our study, six ST78 isolates were distributed among four clinics (UVC, clinic-3, clinic-6, and clinic-10), and repeated isolation was only shown for clinic-3. This is consistent with findings from humans, where this major *E. cloacae* ST exhibits sporadic and clonal distribution (Fernandez et al., 2015; Izdebski et al., 2015, 2017).

Our data show for the first time that the clonal distribution of different genotypes, such as ST11- and ST15-*K. pneumoniae*, ST1196-*E. coli*, and ST506-*E. cloacae*, may significantly contribute to the spread of OXA-48 in companion animals. On the other hand, the identification of several additional sequence types and/or different *Enterobacteriaceae* species confirms previous reports about the high transfer efficiency of a broad host range 62-kb IncL plasmid harboring the *bla*_OXA-48_ genes located within a composite transposon, namely Tn*1999* (Poirel et al., 2012b; Mairi et al., 2017). Insertion of Tn*1999* into the *tir* gene, encoding a transfer inhibition protein, was involved in the higher transfer frequency of the plasmid pOXA-48a (Potron et al., 2014). In our strain collection, *bla*_OXA-48_ was always located on a ∼60-kb plasmid. Sequencing of nine isolates revealed a highly conserved *bla*_OXA-48_-carrying plasmid, which was almost identical to the archetypal pOXA-48a, that contained Tn*1999.2* (Poirel et al., 2012b). This very much resembles the situation in humans, where the emergence of OXA-48 among *Enterobacteriaceae* is mainly related to the spread of one single plasmid.

## Conclusion

We report the emergence of OXA-48 producing *Enterobacteriaceae* in companion animals and pets, which is related to the clonal spread of a few STs and the horizontal dissemination of a single pOXA-48a-like plasmid. We have indications for transmission of CPE isolates within at least two veterinary clinics and suggest that such strains and/or the OXA-48 plasmid can also be transferred between clinics by animal carriers. Most importantly, significant overlaps between human and animal strains could be revealed in terms of bacterial sequence types and the OXA-48 plasmid. Since reports about OXA-48-producing *Enterobacteriaceae* in animals appeared several years after initial findings of these bacteria in humans in the study area, there might be a spillover of OXA-48 plasmids and/or OXA-48 producing *Enterobacteriaceae* from humans to companion animals. However, further phylodynamic studies based on whole genome sequencing would be necessary to verify this hypothesis and to understand possible transmission pathways more properly A systematic monitoring is urgently needed to assess the dissemination of CPE not only in livestock but particularly in companion animals and veterinary clinics on a global scale.

## Author Contributions

CE and EP-B planned the study. SP, InS, IvS, EP-B, and UL identified the isolates and conducted the antimicrobial susceptibility and resistance gene testing. SP, InS, and UL performed transconjugation analysis and PFGE. Data analysis was carried out by SP, InS, CE, and CH. CE and TS performed plasmid sequence data analysis. CE and InS wrote the manuscript. All authors read and approved the final manuscript.

## Conflict of Interest Statement

The authors declare that the research was conducted in the absence of any commercial or financial relationships that could be construed as a potential conflict of interest.

## Footnotes

1 http://www.eucast.org/ast_of_bacteria/guidance_documents/

2 http://bigsdb.pasteur.fr/klebsiella/klebsiella.html

3 http://bigsdb.pasteur.fr/ecoli/ecoli.html

4 https://pubmlst.org/koxytoca/

5 https://pubmlst.org/ecloacae/

6 http://cab.spbu.ru/software/spades/

7 http://rast.nmpdr.org/"

8 https://blast.ncbi.nlm.nih.gov/Blast.cgi

9 http://www.geneious.com

10 http://brig.sourceforge.net/
