# Supplementary material for: Multispecies and Clonal Dissemination of OXA-48 Carbapenemase in Enterobacteriaceae From Companion Animals in Germany, 2009—2016
Source: Front Microbiol. 2018 Jun 14;9:1265. doi: 10.3389/fmicb.2018.01265 (PMC6010547; doi:10.3389/fmicb.2018.01265)
Supplement: Supplementary file 2 [file Table_2.DOCX]

**Supplemental Table 2:**

**Primers used for mapping of OXA-48 plasmids from animal Enterobacteriaceae isolates by PCR.**

| PCR | Target region | Primer name | 5`-3`sequence | Amplicon size in pOXA-N1 (Acc.-no. NC_021488) | Annealing Temperature (° C) | Primer reference |
| --- | --- | --- | --- | --- | --- | --- |
| 1 | *ssb* | ssb(pOXA-48)-FP | ACGTAACACCAGTGCAGAAG | 1658 bp | 57 | This study |
|  | *mobC* | mobC (pOXA-48)-RP | CTCAGAATCCTGACGCTTCA |  |  |  |
| 2 | *trbN* | trbN(pOXA-48)-FP | AATCCACGGAGAGGAATGTG | 1159 bp | 57 | This study |
|  | *tnpA* | tnpA(pOXA-48)-RP | GCCTGGGTATTATTCGCCAA |  |  |  |
| 3 | *tnpA* | tnpA(pOXA-48)-FP | AGCTGCATGACAAAGTCATCG | 1306 bp | 57 | This study |
|  | *mcmM* | mcmM(pOXA-48)-RP | CATCGTCCAGTTGTACCTCGA |  |  |  |
| 4 | *mobC* | mobC-FP | AATCGCCATGCCGAACCGG | 2997 bp | 61 | This study |
|  | *traH* | traH-RP | CAGCTGCAGTTCACCGGGTT |  |  |  |
| 5 | *traH* | traH-FP | TCATTCCTCAGAGGGACCCG | 2639 bp | 61 | This study |
|  | *primase gene* | DNA-Primase-RP | CTGCAGGTCGGCCATCCAGA |  |  |  |
| 6 | *primase gene* | DNA-Primase-FP | TACACCGCACCAACAGATCG | 2192 bp | 60 | This study |
|  | *traL* | traL-RP | CACCACTTCCACTCGCAGCT |  |  |  |
| 7 | *traL* | traL-FP | ACAGTGATGCATTCCCGTTC | 2629 bp | 57 | This study |
|  | *traO* | traO-RP | CCACCGACGTAGAGCATCAT |  |  |  |
| 8 | *traO* | traO-FP | TCAGCAGATGCCTCAGCCTC | 2922 bp | 61 | This study |
|  | *traU* | traU-B | TCGCGTCATGCGTGATCTTC |  |  | [[1](#_ENREF_1)] |
| 9 | *traU* | traU-FP | ACTATGGCGATGAAGCTCGC | 2335 bp | 61 | This study |
|  | *traY* | traY-RP | CTCGCTAGTTCCGACGCTGT |  |  |  |
| 10 | *traY* | traY-FP | ACAGGTGGACGTCCTTCTGG | 2303 bp | 61 | This study |
|  | *repA* | RepA-B | CGTGCAGTTCGTCTTTCGGC |  |  | [[1](#_ENREF_1)] |
| 11 | *repA* | repA-FP | AGTACGCAGCCACCTGTATC | 3148 bp | 57 | This study |
|  | *trbB* | trbB-RP | CTGAACATGACGGACCTGCT |  |  |  |
| 12 | *trbB* | trbB-FP | TCCCGGTATCGAGTACAGGAG | 2392 bp | 57 | This study |
|  | *trbN* | trbN-RP | CTGCCAACTTGCATGGTTGA |  |  |  |
| 13 | *trbN* | trbN(pOXA-48)-FP | AATCCACGGAGAGGAATGTG | 3828 bp | 57 | This study |
|  | *bla_OXA-48_* | praeOXA-48-RP | CCTAGAAGTGGTTAGCGCGTA |  |  |  |
| 14 | *hyp. prot. gene* | hypProt-B-FP | TGAGCATCCGTCACTGCAGC | 1893 bp | 61 | This study |
|  | *korC* | korC-RP | CCACGTTAAGCGGCACGGT |  |  |  |
| 15 | *hyp. prot. gene* | hypProt-A-FP | TCGAGAGTTATAAGCAGCGC | 2597 bp | 57 | This study |
|  | *rmoA* | rmoA-RP | CCCGCATGGATTCGTACATT |  |  |  |
| 16 | *rmoA* | rmoA-FP | AATGTACGAATCCATGCGGG | 3056 bp | 61 | This study |
|  | *ssb* | ssb-RP | GCTCGCAATGGCCGTTCTGT |  |  |  |
| 17 | *trbN* | trbN(pOXA-48)-FP | AATCCACGGAGAGGAATGTG | 2878 bp | 58 | This study |
|  | *lysR* | lysR-RP | CATCATCATGGGTACTCTCGAT |  |  |  |
| 18 | *lysR* | lysR-FP | TCGAGCCGCAGACAATTAGC | 3367 bp | 59 | This study |
|  | *mccm* | mccm(pOXA-48)-RP | CATCGTCCAGTTGTACCTCGA |  |  |  |
| 19 | *tnpA* | tnpA(pOXA-48)-FP | GCCTGGGTATTATTCGCCAA | 1536 bp | 58 | This study |
|  | *mucB* | mucB-RP | GCTGCAGTACATCGAATTGACA |  |  |  |
| 20 | *mucB* | mucB-FP | TGATCCGGAATATGCCATGC | 3372 bp | 57 | This study |
|  | *hyp. prot. gene* | HypProt-C-RP | CCTTGTACGCTTCATATTCCA |  |  |  |
| 21 | *hyp. prot. gene* | HypProt-C-FP | TGGAATATGAAGCGTACAAGG | 2230 bp | 57 | This study |
|  | *hyp. prot. gene* | HypProt-D-RP | GCGCTGCATATACCCTGAAT |  |  |  |
| 22 | *hyp. prot. gene* | HypProt-D-FP | ATTCAGGGTATATGCAGCGC | 2835 bp | 58 | This study |
|  | *parA* | ParA-RP | CGCATCTCTGCTGACGTGTA |  |  |  |

**References**

1. Poirel, L., R.A. Bonnin, and P. Nordmann, *Genetic features of the widespread plasmid coding for the carbapenemase OXA-48.* Antimicrob Agents Chemother, 2012. **56**(1): p. 559-62.
