# Supplementary material for: Multispecies and Clonal Dissemination of OXA-48 Carbapenemase in Enterobacteriaceae From Companion Animals in Germany, 2009—2016
Source: Front Microbiol. 2018 Jun 14;9:1265. doi: 10.3389/fmicb.2018.01265 (PMC6010547; doi:10.3389/fmicb.2018.01265)
Supplement: Supplementary file 3 [file Presentation_1.PPTX]

## Slide 1
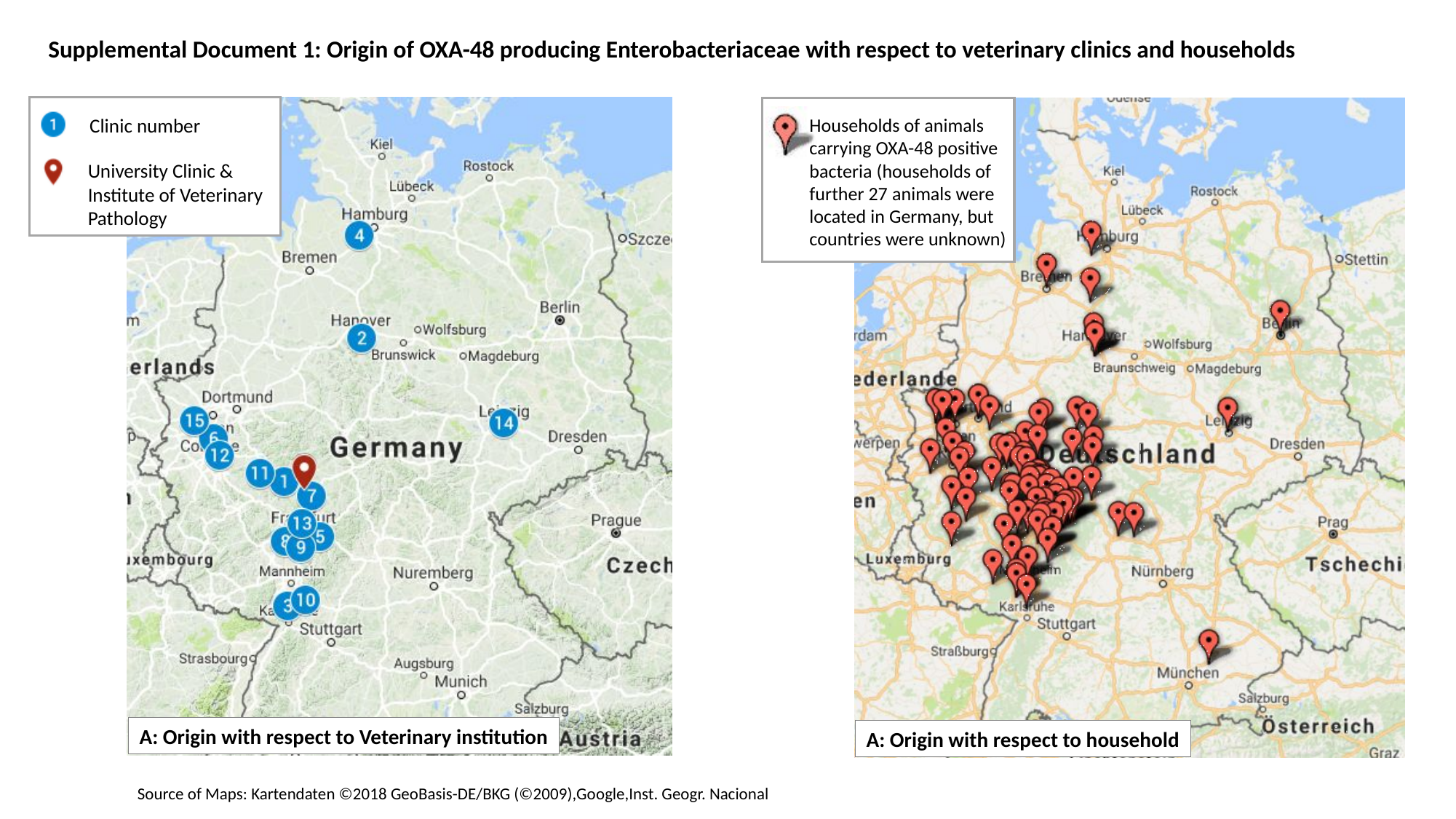

Supplemental Document 1: Origin of OXA-48 producing Enterobacteriaceae with respect to veterinary clinics and households
Clinic number
University Clinic &
Institute of Veterinary Pathology
Households of animals carrying OXA-48 positive bacteria (households of further 27 animals were located in Germany, but countries were unknown)
A: Origin with respect to Veterinary institution
A: Origin with respect to household
Source of Maps: Kartendaten ©2018 GeoBasis-DE/BKG (©2009),Google,Inst. Geogr. Nacional
