# Supplementary figures and images for: Multispecies and Clonal Dissemination of OXA-48 Carbapenemase in Enterobacteriaceae From Companion Animals in Germany, 2009—2016
Source: Front Microbiol. 2018 Jun 14;9:1265. doi: 10.3389/fmicb.2018.01265 (PMC6010547; doi:10.3389/fmicb.2018.01265)

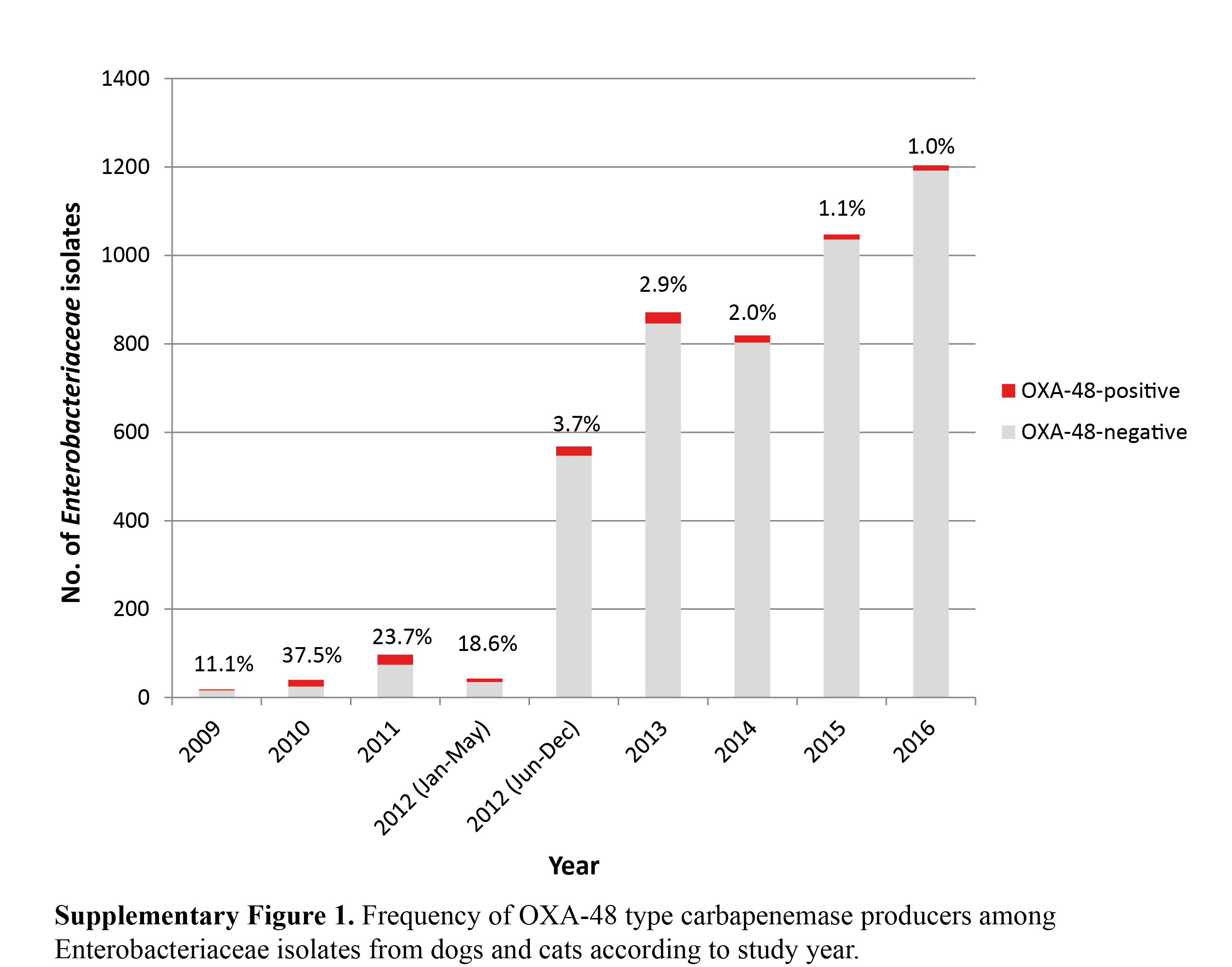

Supplement: Supplementary file 4 [file Image_1.TIF]
