# Supplementary material for: Multispecies and Clonal Dissemination of OXA-48 Carbapenemase in Enterobacteriaceae From Companion Animals in Germany, 2009—2016
Source: Front Microbiol. 2018 Jun 14;9:1265. doi: 10.3389/fmicb.2018.01265 (PMC6010547; doi:10.3389/fmicb.2018.01265)
Supplement: Supplementary file 5 [file Image_2.PDF]

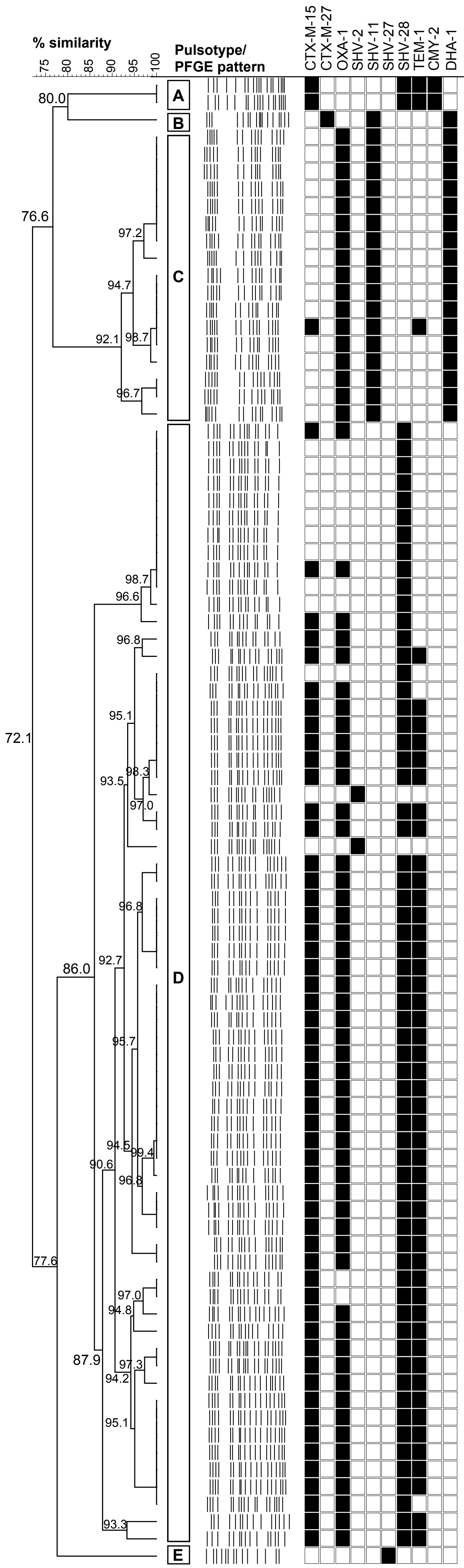

Strain	Clinic origin	ST	Isolation date	Patient	Plasmids (kb)
IHIT30306*	UVC	ST307	20.10.2015	Dog-95	60, 100, 140
IHIT31424*	UVC	ST307	28.04.2016	Dog-97	60, 100, 130, 180
IHIT24175	UVC	ST895	11.11.2013	Dog-73	60, 200
IHIT24179	UVC	ST11	06.09.2013	Dog-70	55, 60
IHIT25327*	UVC	ST11	20.01.2014	Dog-78	55, 60
IHIT25323	UVC	ST11	02.01.2014	Dog-76	55, 60
IHIT23257*	UVC	ST11	01.07.2013	Dog-64	55, 60
IHIT24178	UVC	ST11	24.07.2013	Dog-66	55, 60
IHIT34188	UVC	ST11	09.05.2011	Dog-21	55, 60
IHIT34191	UVC	ST11	01.06.2011	Dog-24	55, 60
IHIT24169	UVC	ST11	06.09.2013	Dog-70	55, 60
IHIT34996	UVC	ST11	22.08.2011	Dog-29	55, 60
IHIT34192	IVP	ST11	06.08.2011	Dog-28	55, 60
IHIT25324	UVC	ST11	02.01.2014	Dog-78	55, 60
IHIT34189	UVC	ST11	18.05.2011	Dog-22	55, 60
IHIT25361	UVC	ST11	21.02.2014	Dog-78	55, 60
IHIT33899	UVC	ST11	04.04.2011	Dog-19	55, 60
IHIT22715*	UVC	ST11	22.10.2012	Dog-52	55, 60, 200
IHIT22717*	UVC	ST11	18.01.2013	Dog-59	55, 60, 200
IHIT33898	UVC	ST11	25.03.2011	Dog-18	55, 60
IHIT24172	UVC	ST15	11.11.2013	Cat-3	60, 100, 120
IHIT24181	UVC	ST15	19.06.2013	Dog-62	30, 50, 60, 100, 150
IHIT24183	UVC	ST15	25.06.2013	Dog-63	30, 50, 60, 100, 150
IHIT24184	UVC	ST15	17.07.2013	Dog-65	30, 50, 60, 100, 150
IHIT24185	UVC	ST15	06.08.2013	Dog-67	30, 50, 60, 100, 150
IHIT24186	UVC	ST15	19.08.2013	Dog-68	30, 50, 60, 100, 150
IHIT24187	UVC	ST15	19.08.2013	Dog-69	30, 50, 60, 100, 150
IHIT24188	UVC	ST15	01.10.2013	Dog-71	30, 50, 60, 100, 150
IHIT25322	UVC	ST15	02.01.2014	Dog-77	60, 100, 200
IHIT25328*	UVC	ST15	20.01.2014	Dog-79	30, 50, 60, 100, 150
IHIT24182	UVC	ST15	10.07.2013	Dog-64	30, 50, 60, 100, 150
IHIT24177*	UVC	ST15	19.11.2013	Cat-4	60, 100, 180
IHIT24171*	UVC	ST15	18.10.2013	Dog-72	60, 100, 180
IHIT35000	UVC	ST15	07.10.2011	Dog-32	50, 60, 150, 180
IHIT23256*	UVC	ST15	06.06.2013	Dog-61	30, 50, 60, 100, 150
IHIT24170	UVC	ST15	18.10.2013	Dog-72	60, 100, 180
IHIT24670*	UVC-	ST15	24.08.2009	Dog-1	50, 60, 150, 180
IHIT24676	UVC	ST15	03.08.2010	Dog-7	50, 60, 150, 180
IHIT24679	IVP	ST15	13.08.2010	Guinea pig	50, 60, 150, 180
IHIT24686*	IVP	ST15	14.10.2010	Mouse-1	50, 60, 150, 180
IHIT34187	UVC	ST15	09.05.2011	Dog-20	50, 60, 150, 180
IHIT24677	UVC	ST15	06.08.2010	Dog-8	50, 60, 100, 180
IHIT24685	Clinic-1	ST15	04.10.2010	Dog-14	60, 150, 180
IHIT25373	UVC	ST15	18.03.2014	Dog-80	60, 100, 200
IHIT24684*	IVP	ST15	13.09.2010	Rabbit-1	40, 60, 100, 180
IHIT22063*	UVC	ST15	22.08.2012	Dog-48	50, 60, 75, 100
IHIT22065*	UVC	ST15	25.09.2012	Dog-49	50, 60, 75, 100
IHIT22061*	UVC	ST15	12.06.2012	Dog-47	60, 130, 290
IHIT22719	UVC	ST15	24.10.2012	Dog-52	50, 60, 75, 100
IHIT22721*	UVC	ST15	01.10.2012	Dog-49	50, 60, 75, 100
IHIT22722	UVC	ST15	02.01.2013	Dog-57	50, 60, 75, 100
IHIT22723	UVC	ST15	02.01.2013	Dog-58	50, 60, 75, 100
IHIT24671	UVC	ST15	21.08.2009	Dog-2	30, 60, 150
IHIT35008	UVC	ST15	05.03.2012	Dog-40	50, 60, 75
IHIT24682	IVP	ST15	13.09.2010	Rat-1	60, 160, 180
IHIT34993	UVC	ST15	06.07.2011	Dog-25	50, 60, 75
IHIT34994	UVC	ST15	20.07.2011	Dog-26	50, 60, 150
IHIT34995	UVC	ST15	27.07.2011	Dog-27	50, 60, 75
IHIT34999	UVC	ST15	11.10.2011	Dog-31	50, 60, 75
IHIT35004	UVC	ST15	09.11.2011	Dog-35	50, 60, 75
IHIT35003	UVC	ST15	21.11.2011	Dog-34	50, 60, 75, 100
IHIT35006	UVC	ST15	23.11.2011	Dog-37	50, 60, 75, 100
IHIT35005	UVC	ST15	01.12.2011	Dog-36	50, 60, 75, 100
IHIT33540	Clinic-15	ST15	10.2016	Cat-13	60, 100, 140
IHIT22059*	UVC	ST15	11.06.2012	Dog-42	50, 60, 75, 100
IHIT34197	UVC	ST15	31.08.2012	Dog-45	50, 60, 75, 100
IHIT34190	UVC	ST15	26.06.2011	Dog-23	50, 60, 75
IHIT34998	UVC	ST15	04.10.2011	Dog-30	50, 60, 75, 100
IHIT34997	UVC	ST15	10.10.2011	Dog-30	50, 60, 75, 100
IHIT27786	UVC	ST15	21.10.2014	Dog-86	50, 60, 130, 190
IHIT27787	Clinic-7	ST15	06.11.2014	Dog-86	50, 60, 130, 190
IHIT24675	UVC	ST15	28.05.2010	Dog-5	60, 150, 230
IHIT24673	UVC	ST15	11.05.2010	Cat-1	60, 150, 180
IHIT35001	UVC	ST15	17.10.2011	Dog-33	50, 60, 150, 180
IHIT35002	UVC	ST15	07.11.2011	Dog-33	50, 60, 150, 180
IHIT24681	UVC	ST15	18.08.2010	Dog-11	60, 160, 180
IHIT24674	UVC	ST15	20.05.2010	Dog-4	40, 60, 150, 180
IHIT24678	UVC	ST15	12.08.2010	Dog-9	40, 60, 150, 180
IHIT24680	UVC	ST15	13.08.2010	Dog-10	40, 60, 150, 180
IHIT24683	UVC	ST15	13.09.2010	Dog-12	40, 60, 150, 180
IHIT24673	UVC	ST15	23.03.2010	Dog-3	60, 150, 180
IHIT24687	UVC	ST15	11.11.2010	Dog-15	40, 60, 150, 180
IHIT25059	UVC	ST15	11.02.2014	Dog-80	60, 100, 200
IHIT22066*	UVC	ST15	06.10.2012	Dog-52	50, 60, 75, 100
IHIT22720	UVC	ST15	07.12.2012	Dog-56	50, 60, 75, 100
IHIT32318	UVC	ST661	04.08.2016	Cat-10	60

Supplemental Figure 2A. Pulsed-field gel electrophoresis grouping, beta-lactamase types, multilocus sequence types (ST), plasmid profiles and origin of OXA-48-producing *Klebsiella pneumoniae* isolates. Dogs, cats, and other animal species were each numbered chronologically according to the time of sampling. Strains marked with an asterik (*) were selected for transconjugation assays described in the main text.
