# Supplementary material for: Multispecies and Clonal Dissemination of OXA-48 Carbapenemase in Enterobacteriaceae From Companion Animals in Germany, 2009—2016
Source: Front Microbiol. 2018 Jun 14;9:1265. doi: 10.3389/fmicb.2018.01265 (PMC6010547; doi:10.3389/fmicb.2018.01265)
Supplement: Supplementary file 6 [file Image_3.PDF]

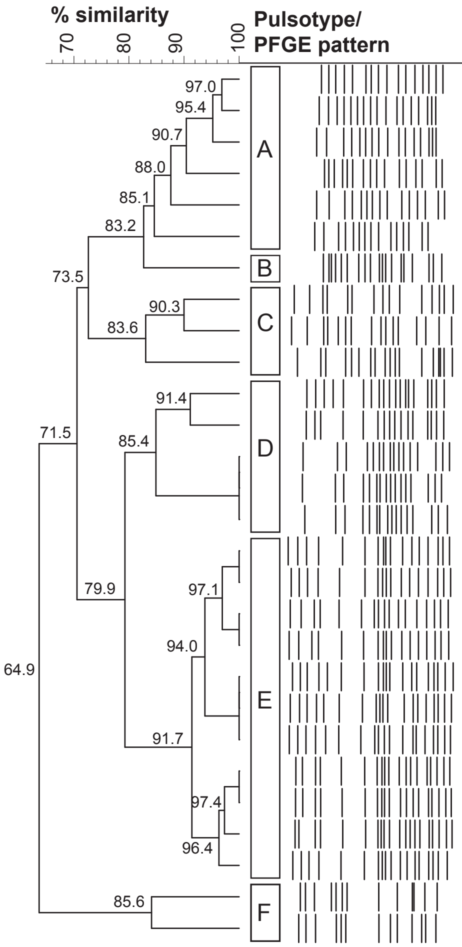


| Pulsotype | CTX-M-15 | OXA-1 | SHV-1 | SHV-12 | TEM-1 | DHA-1 | Strain | Clinic origin | ST | Isolation date | Patient | Plasmids (kb) |
|---|---|---|---|---|---|---|---|---|---|---|---|---|
| A | | | | | | ■ | IHIT30026* | UVC | ST78 | 01.09.2015 | Dog-94 | 40, 60, 110, 170 |
| A | | | | | | | IHIT32761 | Clinic-3 | ST78 | 03.09.2016 | Cat-11 | 40, 60, 70, 85, 110 |
| A | | | | | | | IHIT28624 | Clinic-3 | ST78 | 03.02.2015 | Dog-89 | 40, 60, 70, 85, 110 |
| A | | | | | | | IHIT26632* | Clinic-6 | ST78 | 28.07.2014 | Cat-5 | 40, 60, 100 |
| A | | | | | | | IHIT23114* | Clinic-3 | ST78 | 25.03.2013 | Dog-60 | 40, 60, 95 |
| A | | | | | | | IHIT29601* | Clinic-10 | ST78 | 23.06.2015 | Dog-91 | 40, 60, 95 |
| B | | | | | | | IHIT29865* | Clinic-11 | ST265 | 12.08.2015 | Dog-92 | 60, 180 |
| C | | | | | ■ | | IHIT25894* | Clinic-5 | ST418 | 07.05.2014 | Dog-84 | 30, 60, 65, 90 |
| C | | | | | ■ | | IHIT28495* | UVC | ST418 | 30.12.2014 | Dog-88 | 40, 60, 75, 100 |
| C | | | | | ■ | | IHIT29256* | Clinic-3 | ST418 | 06.05.2015 | Cat-7 | 60, 75, 100 |
| D | | | | ■ | ■ | | IHIT24291* | Clinic-4 | ST114 | 29.11.2013 | Dog-75 | 60, 250 |
| D | | | | ■ | ■ | ■ | IHIT32369 | Clinic-14 | ST114 | 10.2016 | Dog-103 | 60, 290 |
| D | | | | | ■ | | IHIT27937* | Clinic-8 | ST507 | 09.12.2014 | Cat-6 | 60, 110 |
| D | | | | | ■ | | IHIT27939* | Clinic-9 | ST507 | 09.12.2014 | Dog-87 | 60, 110 |
| D | | | | | ■ | | IHIT33427 | Clinic-13 | ST507 | 23.12.2016 | Cat-12 | 60, 110 |
| E | | | | ■ | ■ | | IHIT29093 | IVP | ST506 | 13.06.2012 | Dog-43 | 60, 100, 180, 290 |
| E | | | | ■ | ■ | | IHIT29094 | UVC | ST506 | 13.08.2012 | Dog-44 | 60, 200, 290 |
| E | | ■ | ■ | | | | IHIT33897 | UVC | ST506 | 17.01.2011 | Dog-17 | 40, 60, 75, 180 |
| E | | | | ■ | ■ | | IHIT29092 | UVC | ST506 | 24.02.2012 | Dog-38 | 60, 200, 290 |
| E | | | | ■ | ■ | | IHIT24721* | UVC | ST506 | 15.01.2014 | Dog-80 | 60, 200, 290 |
| E | | | | ■ | ■ | | IHIT25060 | UVC | ST506 | 20.02.2014 | Dog-81 | 60, 200, 290 |
| E | | | | ■ | ■ | | IHIT27279* | UVC | ST506 | 12.08.2014 | Dog-85 | 60, 200, 290 |
| E | | | | ■ | ■ | | IHIT28629* | UVC | ST506 | 28.07.2010 | Dog-6 | 60, 200, 290 |
| E | | | | ■ | ■ | | IHIT28630* | UVC | ST506 | 22.09.2010 | Dog-13 | 60, 200, 290 |
| E | | | | ■ | ■ | | IHIT28631* | UVC | ST506 | 09.11.2010 | Dog-16 | 60, 100, 200, 290 |
| E | | | | ■ | ■ | | IHIT24150 | UVC | ST506 | 29.10.2013 | Dog-74 | 60, 180, 290 |
| F | | | | | ■ | | IHIT29387* | UVC | ST124 | 10.06.2015 | Cat-8 | 60 |
| F | ■ | ■ | | | ■ | | IHIT29776* | UVC | ST124 | 02.07.2015 | Cat-9 | 60, 350 |

**Supplemental Figure 2B.** Pulsed-field gel electrophoresis grouping, beta-lactamase types, multilocus sequence types (ST), plasmid profiles and origin of OXA-48-producing *Enterobacter cloacae* isolates. Dogs and cats and other animals were each numbered chronologically according to the time of sampling. Strains marked with an asterik (*) were selected for transconjugation assays described in the main text.
