# Supplementary material for: Multispecies and Clonal Dissemination of OXA-48 Carbapenemase in Enterobacteriaceae From Companion Animals in Germany, 2009—2016
Source: Front Microbiol. 2018 Jun 14;9:1265. doi: 10.3389/fmicb.2018.01265 (PMC6010547; doi:10.3389/fmicb.2018.01265)
Supplement: Supplementary file 7 [file Image_4.PDF]

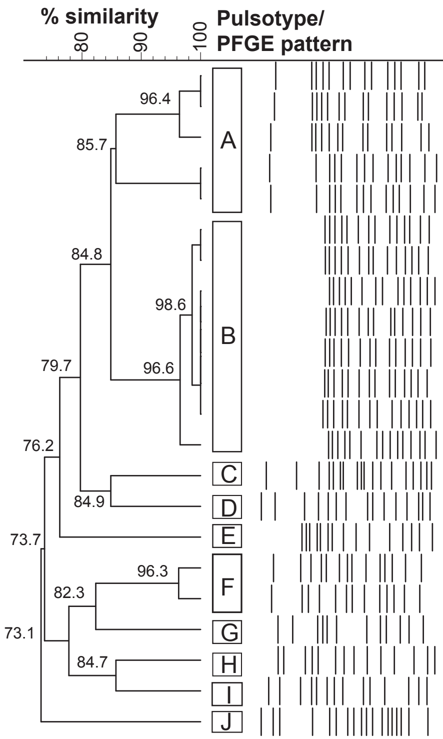

Pulsotype	CTX-M-1	CTX-M-15	OXA-1	SHV-12	TEM-1	CMY-2	Strain	Clinic origin	ST	Isolation date	Patient	Plasmids (kb)
A		■	■		■		IHIT28839	UVC	ST617	25.02.2015	Dog-90	60, 180
A					■		IHIT31866*	UVC	ST617	12.03.2016	Dog-98	60, 80
A					■		IHIT31262*	UVC	ST617	15.03.2016	Dog-96	60, 100
A			■				IHIT32307	UVC	ST88	15.08.2016	Dog-99	60, 100
A			■				IHIT32893	UVC	ST88	21.10.2016	Dog-100	60, 80
B				■	■	■	IHIT34194	UVC	ST1196	08.03.2012	Dog-39	60, 180, 290
B				■	■	■	IHIT34196	UVC	ST1196	03.04.2012	Dog-41	60, 180, 290
B				■	■	■	IHIT22062*	UVC	ST1196	12.06.2012	Dog-47	60, 130, 290
B				■	■	■	IHIT22060*	UVC	ST1196	11.06.2012	Dog-42	60, 180, 290
B				■	■	■	IHIT35007	UVC	ST1196	08.03.2012	Dog-39	60, 180, 290
B				■	■	■	IHIT34195	UVC	ST1196	02.04.2012	Cat-2	60, 180, 290
B				■	■	■	IHIT21159	Clinic-2	ST1196	11.10.2012	Dog-49	60, 180, 290
B					■	■	IHIT22714*	UVC	ST1196	05.11.2012	Dog-53	40, 60, 130
C					■		IHIT33900	UVC	ST345	11.06.2012	Dog-42	60, 80
D	■				■		IHIT22064*	UVC	ST1431	25.09.2012	Dog-50	50, 60, 80, 130
E			■		■		IHIT29873*	Clinic-10	ST58	10.08.2015	Dog-93	60, 160
F					■		IHIT22718*	UVC	ST410	05.11.2012	Dog-54	60, 100
F					■		IHIT35009	UVC	ST410	20.09.2012	Dog-46	60, 80, 100
G		■					IHIT30276*	UVC	ST744	20.10.2015	Dog-95	40, 60, 130
H							IHIT25661*	Clinic-3	ST372	09.04.2014	Dog-83	60
I					■		IHIT33426	Clinic-12	ST783	22.12.2016	Dog-102	50, 60, 130
J		■	■		■		IHIT33076	UVC	ST648	20.10.2016	Dog-101	60, 80, 130

Supplemental Figure 2C. Pulsed-field gel electrophoresis grouping, beta-lactamase types, multilocus sequence types (ST), plasmid profiles and origin of OXA-48-producing *Escherichia coli* isolates. Dogs and cats and other animals were each numbered chronologically according to the time of sampling. Strains marked with an asterik (*) were selected for transconjugation assays described in the main text.
